# Additional Measurement Approaches for Sleep Disturbances. Comment on “A Transdiagnostic Self-management Web-Based App for Sleep Disturbance in Adolescents and Young Adults: Feasibility and Acceptability Study”

**DOI:** 10.2196/35959

**Published:** 2022-06-13

**Authors:** Wan-Tong Tsai, Tzung-Liang Liu

**Affiliations:** 1 Chung Shan Medical University Taichung City Taiwan

**Keywords:** youth, sleep, technology, mHealth, self-management, adolescents, young adults, mobile phone, smartphone, polysomnography

Carmona et al [1] analyzed the effect of DOZE (Delivering Online Zzz's with Empirical Support) on adolescents and young adults. However, this was done only via feedback from the participants. To make measurements more quantitative and less subjective, we propose a few scientific measurement approaches.

Polysomnography [2], a method to measure one’s physiological state during sleep, is a common way to assess sleep quality. With the following devices, 7 parts of the physiological state can be detected:

1. Electroencephalogram: Multiple brain electrode patches are placed on the scalp to record the various stages of sleep. Thus, we can obtain a brainwave diagram for analysis.
2. Electromyography: 4 electrode patches are used to monitor muscle tension. Muscle tension will decrease significantly during sleep. We can also detect whether periodic limb movement disorder occurs and then compare the results with other data.
3. Electrocardiography: this can record the activity of the heart, including T waves, P waves, and QRS waves.
4. Electrooculography: this can record the potential difference between the cornea and the retina, so that we know when the rapid eye movement period occurs, allowing us to judge the stages of sleep.
5. Oxygen saturation: by recording this, we can know if the body remains in good condition during sleep.
6. Thoracic abdominal effort.
7. Nasal and oral airflow.

In addition, a serology examination can also be applied to quantify the survey. For instance, we can detect the melatonin levels of participants, so as to detect fluctuations from baseline to the end point. Thus, we can see if the concentration of melatonin reaches appropriate levels after participants use a web-based self-management app [3].

To keep the self-management app easy to use and convenient, any approach that requires specific tests in a hospital should not be used. Regardless, there are some take-home tests that can be administered easily. Examples include self-adhesive electrodes; heart rate monitoring equipment; or even techniques like the Freestyle Libre Pro for glucose monitoring [4], which can be used to track the physiological state of each participant.

In summary, quantifying the influence of DOZE with physiological states could make it more credible, and monitoring tests can be put into practice feasibly.

## Abbreviations

DOZE: Delivering Online Zzz's with Empirical Support
